# Roles of FGF Signals in Heart Development, Health, and Disease

**DOI:** 10.3389/fcell.2016.00110

**Published:** 2016-10-18

**Authors:** Nobuyuki Itoh, Hiroya Ohta, Yoshiaki Nakayama, Morichika Konishi

**Affiliations:** ^1^Medical Innovation Center, Kyoto University Graduate School of MedicineKyoto, Japan; ^2^Department of Microbial Chemistry, Kobe Pharmaceutical UniversityKobe, Japan

**Keywords:** development, disease, FGF, heart, biomarker, differentiation

## Abstract

The heart provides the body with oxygen and nutrients and assists in the removal of metabolic waste through the blood vessels of the circulatory system. It is the first organ to form during embryonic morphogenesis. FGFs with diverse functions in development, health, and disease are signaling proteins, mostly as paracrine growth factors or endocrine hormones. The human/mouse FGF family comprises 22 members. Findings obtained from mouse models and human diseases with FGF signaling disorders have indicated that several FGFs are involved in heart development, health, and disease. Paracrine FGFs including FGF8, FGF9, FGF10, and FGF16 act as paracrine signals in embryonic heart development. In addition, paracrine FGFs including FGF2, FGF9, FGF10, and FGF16 play roles as paracrine signals in postnatal heart pathophysiology. Although FGF15/19, FGF21, and FGF23 are typical endocrine FGFs, they mainly function as paracrine signals in heart development or pathophysiology. In heart diseases, serum FGF15/19 levels or FGF21 and FGF23 levels decrease or increase, respectively, indicating their possible roles in heart pathophysiology. FGF2 and FGF10 also stimulate the cardiac differentiation of cultured stem cells and cardiac reprogramming of cultured fibroblasts. These findings provide new insights into the roles of FGF signaling in the heart and potential therapeutic strategies for cardiac disorders.

## Introduction

The heart is a muscular organ that pumps blood. It provides the body with oxygen and nutrients and assists in the removal of metabolic waste through the blood vessels of the circulatory system. The heart is the first organ to form during embryonic development. Embryonic heart development requires proper communication between cardiac progenitor cells (Miquero and Kelly, 2013; Meganathan et al., 2015). Heart failure, which is a systemic disorder caused by the inability of the heart to pump blood, represents a major cause of morbidity and mortality and remains a critical health issue (Wilsbacher and McNally, 2016). A number of secreted proteins including bone morphogenetic proteins, insulin-like growth factors, Wnts, vascular endothelial growth factor, and erythropoietin function as paracrine or endocrine signals in heart development, health, and disease (Meganathan et al., 2015; Wilsbacher and McNally, 2016).

Fibroblast growth factors (FGFs) are also secreted signaling proteins. In humans and mice, the FGF family comprises 22 members. Most FGFs play roles as paracrine or endocrine signals in development, health, and disease in major organs including the liver, kidney, brain, and bone (Ornitz and Itoh, 2015; Ornitz and Marie, 2015; Brewer et al., 2016; Itoh et al., 2016; Turner et al., 2016). FGFs also act as paracrine or endocrine signals in heart development, health, and disease. These findings provide new insights into the roles of FGFs in the heart and potential therapeutic strategies for heart disorders. A succinct review of the roles of FGFs in the heart is provided herein.

## The FGF family

The prototypic FGFs, FGF1, and FGF2, are secreted signaling proteins of ~150 amino acids. FGF1 and FGF2, which were originally isolated from the brain as growth factors for fibroblasts, are expressed in various embryonic and adult tissues and exhibit diverse activities in cell proliferation, angiogenesis, neuronal cell growth, and survival, and wound healing. In humans and mice, 22 FGFs of ~150–300 amino acids with a conserved core of ~120 amino acids (~30–60% amino acid identity) have a wide variety of functions in development, health, and disease. However, the human and mouse FGF families do not include FGF15 or FGF19, respectively, because they are orthologs. Since they have been named FGF15 in mice and FGF19 in humans, they are typically referred to as FGF15/19. A phylogenetic analysis has classified FGFs into seven subfamilies based on their possible evolutionary relationships. They have also been classified into paracrine, endocrine, and intracrine FGFs based on their mechanisms of action (**Figure 2**; Ornitz and Itoh, 2015; Brewer et al., 2016).

**Figure 1:**
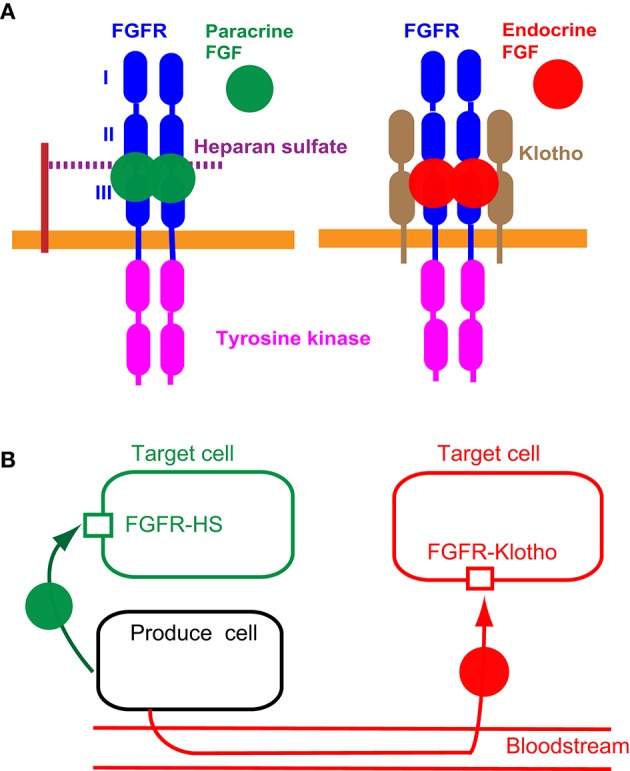
**Mechanisms of action of paracrine and endocrine FGFs. (A)** Paracrine FGFs specifically bind to the FGFR-heparan sulfate complex and activate FGFR tyrosine kinase. Endocrine FGFs specifically bind to the FGFR-Klotho complex and activate tyrosine kinase. This, in turn, induces the activation of intracellular pathways (Ornitz and Itoh, 2015; Brewer et al., 2016). **(B)** Paracrine FGFs are secreted local signals that act on nearby target cells by diffusion with functions in multiple developmental and pathophysiological processes. Endocrine FGFs are secreted endocrine signals that act on distant target cells through the bloodstream with multiple functions in metabolic and pathophysiological processes. FGFR-HS and FGFR-Klotho indicate the FGFR-heparan sulfate complex and FGFR-Klotho complex, respectively (Ornitz and Itoh, 2015; Brewer et al., 2016).

Paracrine FGFs including FGF1-FGF10, FGF16-FGF18, FGF20, and FGF22 are secreted proteins with heparan sulfate-binding sites at their carboxyl termini. Paracrine FGFs exert biological activities by binding to cell surface FGF receptors (FGFRs) with heparan sulfate as a co-factor (Figure 1A). Paracrine FGFs mainly function in multiple developmental and physiological processes by acting on nearby target cells as local signals via diffusion (Figure 1B). Heparan sulfate with long linear carbohydrate chains of repeating sulfated glucuronic acid linked to N-acetylglucosamine disaccharides is covalently linked to specific cell surface transmembrane-type proteins. It modulates paracrine FGF diffusion, the modulation of which directs paracrine FGFs as local signals (Ornitz and Itoh, 2015; Brewer et al., 2016).

Seven major FGFR proteins (FGFRs 1b, 1c, 2b, 2c, 3b, 3c, and 4) are generated from the *FGFR1, FGFR2, FGFR3*, and *FGFR4* genes by alternative splicing. FGFRs have different FGF-binding specificities. Heparan sulfate, which independently interacts with FGFs and FGFRs, is necessary for stable interactions between FGFs and FGFRs. The FGF-FGFR-heparan sulfate complex leads to FGFR dimerization and directs the activation of FGFR intracellular tyrosine kinase domains (Figure 1A), followed by key intracellular signaling pathways including the rat sarcoma protein (RAS)-mitogen-activated protein kinase (MAPK), phosphoinositide 3-kinase (PI3K)-AKT serine/threonine kinase (AKT), phospholipase Cγ (PLCγ), and signal transducer and activator of transcription (STAT) pathways (Carter et al., 2015; Ornitz and Itoh, 2015; Brewer et al., 2016).

**Figure 2:**
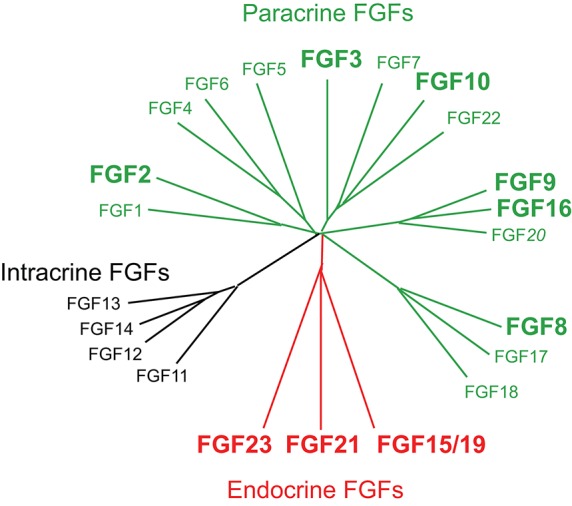
**Evolutionary relationships within the human FGF family**. Phylogenetic analyses suggest that 22 members of the FGF family are classified into several subfamilies. Branch lengths are proportional to the evolutionary distance between each FGF. FGFs are also classified into paracrine, endocrine, and intracrine FGFs based on their mechanisms of action (Ornitz and Itoh, 2015; Brewer et al., 2016). FGF2, FGF3, FGF8, FGF9, FGF10, FGF15/19, FGF16, FGF21, and FGF23 play roles in heart development, health, and disease. This figure is adapted from Itoh et al. (2016).

Endocrine FGFs comprising FGF15/19, FGF21, and FGF23 are also secreted proteins (Figure 2). However, endocrine FGFs do not usually function as local signals due to their reduced heparan sulfate-binding affinity. Endocrine FGFs with Klotho-binding sites at their carboxyl termini require αKlotho or βKlotho as a co-factor for FGFR. Endocrine FGFs mainly function as endocrine signals in multiple metabolic processes (Figure 1A). αKlotho and βKlotho, which are single-pass transmembrane proteins of ~1000 amino acids with a short cytoplasmic domain, share structural similarities and characteristics with each other. They are specifically expressed in the target tissues of endocrine FGFs, which function in an endocrine manner with target-tissue specificity through the bloodstream (Figure 1B; Ornitz and Itoh, 2015). Endocrine FGFs cannot efficiently bind to FGFR, αKlotho, or βKlotho alone; they efficiently bind to the FGFR-Klotho complex. FGF15/19 mostly binds FGFR4 with βKlotho and FGF21 mainly binds FGFR1c with βKlotho. FGF23 mostly binds FGFR1c with αKlotho. This binding, in turn, induces the activation of the intracellular signaling pathways of FGFR. FGF15/19 and FGF23 exhibit metabolic and proliferative activities. However, FGF21 is a unique FGF with metabolic, but not proliferative activity (Itoh et al., 2015; Ornitz and Itoh, 2015). In contrast, intracrine FGFs comprising FGF11-FGF14 are not secreted signals that play roles in the regulation of electrical excitability in neurons in an intracrine manner (Ornitz and Itoh, 2015). Several paracrine and endocrine FGFs are involved in heart development, health, and disease, as indicated by findings obtained from mouse models and human diseases with FGF signaling disorders as described below.

## Developmental roles of FGFs indicated by *FGF* knockout mice

Proper communication between cardiac progenitor cells is required during embryonic heart development. Remodeling of the outflow tract, which is derived from two cardiac progenitor cells, the second heart field and neural crest cells, is essential for the arterial pole. The second heart field is located in the pharyngeal mesoderm, which contributes to the growth of the heart tube during embryonic looping morphogenesis. Neural crest cells, which migrate from the neuroectoderm of the dorsal neural tube, contribute to cushion formation, and dictate the correct septation and alignment of the heart (Miquero and Kelly, 2013; Meganathan et al., 2015). Among FGFs, FGF3, FGF8, FGF9, FGF10, FGF15/19, and FGF16 function as paracrine signals in embryonic heart development (Table 1).

**Table 1:** **Developmental roles of FGFs in the embryonic mouse heart**.

	**Function**	**Heart phenotype**
**PARACRINE SIGNAL**		
FGF8	Loss-of-function	Defect in cardiac looping, the cardiac outflow tract, and anterior heart field development
	Loss-of-function	Decrease in migratory cardiac neural crest survival
FGF9	Loss-of-function	Decrease in cardiomyocyte proliferation
FGF10	Loss-of-function	Decrease in cardiomyocyte proliferation
	Loss-of-function	Decrease in cardiac fibroblast migration
FGF10/FGF3	Loss-of-function	Defect in cardiovascular progenitor cell development
FGF10/FGF8	Loss-of-function	Defect in outflow tract and right ventricle development
FGF15/19	Loss-of-function	Defect in cardiac outflow tract development
FGF16	Loss-of-function	Decrease in cardiomyocyte proliferation
	Loss-of-function	Defect in chamber, atrial, and ventricular walls and trabeculation

### Paracrine signal

#### FGF8

FGF8 as a paracrine signal mainly activates FGFR1c with heparan sulfate as a co-factor in a paracrine manner (Ornitz and Itoh, 2015). *FGF8* is expressed in the early embryonic stages. *FGF8* knockout mice, which lack all embryonic mesoderm and endoderm-derived structures, are lethal at the gastrulation stage (Sun et al., 1999). Findings obtained from *FGF8* knockout mice have also indicated that FGF8 is required for cardiac looping and migratory cardiac neural crest cell survival. Neural crest cell deficiencies result in cardiac outflow tract septation defects (Abu-Issa et al., 2002). FGF8 is also required for anterior heart field development (Ilagan et al., 2006).

Heparan sulfate is a major constituent of the heart extracellular matrix. Exostosin glycosyltransferase 1 (Ext1) is an enzyme that is responsible for the synthesis of heparan sulfate. The outflow tract defect in mesoderm—specific *Ext1* knockout mice has been attributed to the reduced contribution of the second heart field and neural crest cells. Exogenous FGF8 ameliorates defects in the outflow tract and pharyngeal explants, indicating that heparan sulfate modulates FGF8 signaling during early heart development (Zhang R. et al., 2015).

#### FGF9

FGF9 also mainly activates FGFR1c with heparan sulfate as a co-factor in a paracrine manner (Ornitz and Itoh, 2015). *FGF9* knockout mice are lethal shortly after birth due to impaired lung development (Colvin et al., 2001). The embryonic hearts of *FGF9* knockout mice are slightly small. *FGF9* is expressed in the mouse embryonic heart. The proliferation of cardiomyocytes is significantly decreased in the *FGF9* knockout heart at embryonic stages, indicating that FGF9 is a growth factor for embryonic cardiomyocytes (Lavine et al., 2005).

#### FGF10

FGF10 preferentially activates FGFR2b with heparan sulfate as a co-factor in a paracrine manner (Ornitz and Itoh, 2015; Itoh, 2016). *FGF10* knockout mice are lethal shortly after birth due to the lack of multiple organs including limbs and lungs (Sekine et al., 1999). The embryonic hearts of *FGF10* knockout mice also showed that altered ventricular morphology is associated with the impaired proliferation of right, but not left ventricular cardiomyocytes. *FGF10* and *FGFR2b* are both expressed in cardiomyocytes, but not cardiac fibroblasts, indicating that FGF10 regulates regional-specific cardiomyocyte proliferation in the embryonic heart in an autocrine/paracrine manner (Rochais et al., 2014). FGF10 is also essential for the movement of cardiac fibroblasts into the compact myocardium. The inactivation of the FGF10 signaling pathway results in fewer epicardial-derived cells within the compact myocardium, decreased myocardial proliferation, and as a result, a smaller thin-walled heart (Vega-Hernández et al., 2011).

*FGF10* and *FGF3* are also both expressed in or near cardiovascular progenitor cells. The phenotypes of their knockout mice indicate that FGF10 and FGF3 are required for the normal developmental coordination of cardiovascular progenitor cells, but not for their specification (Urness et al., 2011). In addition, *FGF10* and *FGF8* are expressed in the anterior part of the second heart field. The phenotypes of their knockout mice indicate the functional overlap of FGF10 and FGF8 signaling from second heart field mesoderm during development of the outflow tract and right ventricle (Watanabe et al., 2010).

#### FGF15/19

*FGF15/19* is expressed in developing pharyngeal arches. *FGF15/19* knockout mice are gradually lethal from embryonic day 13.5 to postnatal day 7. *FGF15/19* knockout mice have heart defects with malalignment of the aorta and pulmonary trunk. These defects correlate with early morphological defects in the outflow tract, indicating that FGF15/19 is required for proper morphogenesis of the cardiac outflow tract (Vincentz et al., 2005). FGF15/19 typically activates FGFR4 with βKlotho as an endocrine signal (Itoh et al., 2015). However, *FGFR4* knockout mice and β*Klotho* knockout mice, which are alive even during postnatal stages, do not show any heart defects (Yu et al., 2000; Ito et al., 2005). These findings indicate that FGF15/19 plays roles as a paracrine signal in heart development independently of the FGFR4/βKlotho pathway.

#### FGF16

FGF16 mainly activates FGFR1c with heparan sulfate as a co-factor in a paracrine manner (Ornitz and Itoh, 2015). In mouse embryos, *FGF16* is predominantly expressed in cardiomyocytes. *FGF16* knockout mice with the C57 black 6 (C57BL/6) genetic background appear to be normal and fertile. However, heart weight and cardiomyocyte cell numbers are slightly decreased, indicating that FGF16 is a growth factor for embryonic cardiomyocytes. The embryonic heart phenotype is similar to that of *FGF9* knockout mice, suggesting that FGF9 and FGF16 synergistically act on embryonic cardiomyocytes (Hotta et al., 2008). In contrast, *FGF16* knockout mice with the Swiss Black genetic background are lethal at embryonic day 11.5 due to cardiac defects including chamber dilution, thinning of the atrial, and ventricular walls, and poor trabeculation (Lu et al., 2008). These different phenotypes may be due to differences in their genetic backgrounds (Lu et al., 2010).

## Pathophysiological roles of FGFs indicated by *FGF* knockout or transgenic mice

Cardiac remodeling including cardiac hypertrophy and fibrosis subsequently progress to heart failure, which represents a major cause of morbidity and mortality and remains a critical health issue. Cardiomyokines, heart-derived secreted proteins with crucial functions for heart function, may play roles in cardiac remodeling. Cardiomyokines may be therapeutic targets and/or agents of cardiac remodeling (Doroudgar and Glembotski, 2011). Among FGFs, FGF2, FGF9, FGF10, FGF16, and FGF21 have been shown to play pathophysiological roles as cardiomyokines in a paracrine manner in the heart. However, FGF21 has also been shown to function in cardiac protection in an endocrine manner (Table 2).

**Table 2:** **Pathophysiological roles of FGFs in the postnatal mouse heart**.

	**Function**	**Heart phenotype**
**PARACRINE SIGNAL**		
FGF2	Loss-of-function	Protection against induced cardiac hypertrophy and fibrosis Promotion of cardiac ischemia-reperfusion injury
	Gain-of-function	Promotion of cardiac hypertrophy
FGF9	Gain-of-function	Improvement in heart function
	Gain-of-function	Protection against heart injury
FGF10	Gain-of-function	Protection against heart injury
	Gain-of-function	Promotion of cardiomyocyte proliferation
FGF16	Loss-of-function	Promotion of induced cardiac hypertrophy and fibrosis
FGF21	Loss-of-function	Promotion of induced cardiac hypertrophy and inflammation
**ENDOCRINE SIGNAL**		
FGF21	Gain-of-function	Protection against cardiac dysfunction and inflammation
	Loss-of-function	Promotion of cardiac dysfunction and inflammation

### Paracrine signal

#### FGF2

FGF2 mediates biological responses as an extracellular protein by binding to and activating FGFRs with heparan sulfate as a co-factor in a paracrine manner (Ornitz and Itoh, 2015). Although *FGF2* is broadly expressed in mice, *FGF2* knockout mice are viable and appear to be normal. However, myocardial infarct-induced cardiac hypertrophy and fibrosis are protected against in *FGF2* knockout mice (Virag et al., 2007). Isoproterenol-induced cardiac hypertrophy is also protected against in *FGF2* knockout mice (House et al., 2010). Blood renin and angiotensin II levels are increased by two-kidney one-clip (2K1C) in mice, leading to a chronic elevation in blood pressure and compensatory cardiac hypertrophy. 2K1C-induced cardiac hypertrophy and fibrosis is also protected against in *FGF2* knockout mice (Pellieux et al., 2001). In addition, cardiac-specific *FGF2* transgenic mice exhibit exacerbated hypertrophy, and this effect is protected against in the presence of a pharmacological extracellular signal-regulated kinase (ERK) inhibitor (House et al., 2010). These findings indicate that FGF2 promotes cardiac hypertrophy and fibrosis.

In contrast, *FGF2* knockout mice subjected to cardiac ischemia-reperfusion injury have significantly increased myocardial infarct size and worsened cardiac function, indicating that FGF2 protects cardiac ischemia-reperfusion injury (House et al., 2015). The function of FGF2 is mediated by the inhibition of excessive autophagy and increased ubiquitinated protein clearance via the activation of the PI3K-AKT signaling pathway (Wang Z. G. et al., 2015) and also by the suppression of endoplasmic stress and mitochondrial dysfunction via the PI3K-AKT and RAS-MAPK signaling pathways (Wang Z. et al., 2015). The heart expresses *FGFR1c*, which may be effectively activated by FGF2 (Fon Tacer et al., 2010; Ornitz and Itoh, 2015). These findings also indicate that FGF2 acts on cardiac cells in a paracrine manner and promotes cardiac remodeling by activating the RAS-MAPK and PI3K pathways through the activation of FGFR1c.

#### FGF9

Myocardium-specific transgenic *FGF9* in mice stimulates left ventricular hypertrophy with microvessel expansion and preserves systolic and diastolic functions. After myocardial infarction, transgenic *FGF9* enhances hypertrophy in the noninfarcted left ventricular myocardium with increased microvessel density, reduces interstitial fibrosis, attenuates fetal gene expression, and improves systolic function and heart failure mortality. However, in cultured rat cardiomyocytes, FGF9 stimulates the proliferation and network formation of endothelial cells, but does not directly induce hypertrophic effects (Korf-Klingebiel et al., 2011).

Monocytes, key mediators of inflammation, differentiate into pro-inflammatory M1 macrophages, and anti-inflammatory M2 macrophages upon the infiltration of damaged tissue. A FGF9 treatment of an infarcted myocardium in *db/db* diabetic mice results in significantly decreased monocyte infiltration, increased M2 macrophage differentiation and associated anti-inflammatory cytokines, reduced adverse remodeling, and improved cardiac function. FGF9 possesses novel therapeutic potential due to its ability to mediate monocyte to M2 macrophage differentiation and confer cardiac protection in the post-myocardial infarction diabetic heart (Singla et al., 2015).

#### FGF10

Neonatal mouse hearts have the capacity to regenerate. However, this capacity quickly decreases after postnatal day 7. The overexpression of *FGF10* in the myocardium of inducible transgenic mice enhances the expansion of epicardial cells after heart injury through increased proliferation. However, this expansion does not lead to increased epithelial-to-mesenchymal transition or affect fibroblast formation or fibrosis after heart injury (Rubin et al., 2013). Cardiomyocyte proliferation gradually decreases during embryogenesis. In *FGF10* transgenic mice, the cell-cycle re-entry of cardiomyocytes, but not cardiac fibroblasts is promoted, indicating that FGF10 triggers the cell-cycle re-entry of cardiomyocytes in adults, and is, thus, a potential target for cardiac repair (Rochais et al., 2014).

#### FGF16

Cardiac hypertrophy and fibrosis are induced by angiotensin II. Transforming growth factor-β1 (TGF-β1) is a downstream factor of angiotensin II for cardiac hypertrophy and fibrosis. Angiotensin II-induced cardiac hypertrophy and fibrosis are significantly promoted by increasing the expression of *TGF*-β*1* in *FGF16* knockout mice (Matsumoto et al., 2013). The response to cardiac remodeling in *FGF16* knockout mice is apparently opposite to that in *FGF2* knockout mice as described above (Pellieux et al., 2001).

In the heart, *FGF16* and *FGF2* are mainly expressed in cardiomyocytes and non-cardiomyocytes, respectively. FGF16 is a cardiomyocyte-derived paracrine signal (Hotta et al., 2008). FGF2 stored in non-cardiomyocytes is released in response to hemodynamic stress (Kaye et al., 1996). FGF2 exhibits significant proliferative activity in cultured cardiomyocytes, where FGF16 does not. However, FGF16 inhibits the activity of FGF2 by competing with FGF2 for FGFR1c, which is predominantly expressed in the heart (Lu et al., 2008). Furthermore, FGF2 significantly induces the expression of *TGF*-β*1* in cultured cardiomyocytes and non-cardiomyocytes, where FGF16 does not. However, FGF16 inhibits the FGF2-induced expression of *TGF*-β*1*. The biochemical properties of FGF16 are distinct from those of FGF2. Cardiac *FGF16* expression is induced after that of *FGF2* by angiotensin II. *FGF16* expression is promoted by FGF2 in cultured cardiomyocytes. These findings indicate that FGF16 prevents cardiac hypertrophy and fibrosis by competing with FGF2 for FGFR1c (Itoh and Ohta, 2013; Matsumoto et al., 2013; Wang J. et al., 2015).

GATA binding protein (GATA) family members are zinc finger transcription factors. GATA4 regulates a number of cardiac-specific genes that are important for embryonic and neonatal heart development. Ventricular function accompanied by reduced cardiomyocyte replication in the heart is severely depressed in inducible cardiomyocyte-specific *GATA4* knockout mice after injury. After injury, the mutant hearts also display impaired coronary angiogenesis and increased hypertrophy and fibrosis with a significant reduction in the expression of *FGF16*. The cardiac-specific overexpression of *FGF16* in the mutant hearts partially rescues cardiac hypertrophy, promotes cardiomyocyte replication, and improves heart function after injury, indicating that GATA4 is required for neonatal heart regeneration through the regulation of FGF16, which has potential in promoting myocardial repair (Yu et al., 2016).

#### FGF21

FGF21 functions in an FGFR-dependent manner. However, FGF21 binds to FGFRs with heparan sulfate with very low affinity. FGF21 efficiently activates FGFR1c with βKlotho as a co-factor. FGF21 acts on long-distance targeted cells through blood cells in an endocrine manner. *FGF21* is mainly expressed in the liver. The phenotypes of *FGF21* knockout and transgenic mice suggest the diverse metabolic actions of FGF21 in glucose and lipid metabolism in an endocrine manner (Itoh et al., 2015; Kharitonenkov and DiMarchi, 2015). However, FGF21 acts s as a paracrine signal in the heart as described below.

An increased relative heart weight and enhanced signs of dilatation are observed in *FGF21* knockout mice. Cardiac hypertrophy is also more enhanced in response to the infusion of isoproterenol in *FGF21* knockout mice. FGF21 reverses cardiac hypertrophy in *FGF21* knockout mice and cultured cardiomyocytes. Although cardiomyocytes produce FGF21, cardiac FGF21 secretion is lower than hepatic FGF21 secretion. However, FGF21 is abundantly secreted by cardiac cells in response to cardiac stress, and cardiac FGF21 secretion inhibits isoproterenol-induced cardiac hypertrophic damage. In addition, *FGFR1c* and β*Klotho* are both predominantly expressed in cardiomyocytes. These findings indicate that FGF21 acts on cardiomyocytes, possibly in a paracrine manner, and prevents cardiac hypertrophy by activating MAPK signaling through the activation of FGFR1c with βKlotho (Planavila et al., 2013, 2015a).

Oxidative stress by reactive oxygen species plays a prominent role in the pathogenesis of heart failure, and antioxidants attenuate cardiac remodeling. The expression of antioxidant genes in response to the lipopolysaccharide (LPS)-induced stimulation of pro-inflammatory pathways or isoproterenol-induced cardiac hypertrophy is decreased in the hearts of *FGF21* knockout mice. In cultured cardiomyocytes, FGF21 induces the expression of genes involved in antioxidative pathways. FGF21 released by cardiomyocytes in response to LPS acts in an autocrine/paracrine manner to protect cells against oxidative stress. These findings indicate that FGF21, as an autocrine/paracrine signal, prevents induction of pro-oxidative pathways in the heart (Di Lisa and Itoh, 2015; Planavila et al., 2015a,b).

Cardiac *FGF21* expression is increased in a mouse model of type 1 diabetes. FGF21 knockout mice are more susceptible to diabetes-induced cardiac apoptosis, and this may be prevented by the administration of FGF21. FGF21 significantly reduces palmitate-induced cardiac apoptosis in cultured cardiomyocytes. Palmitate suppresses, whereas FGF21 promotes the MAPK pathway. These findings indicate that FGF21 prevents lipid- or diabetes-induced cardiac apoptosis by activating the MAPK pathway (Zhang C. et al., 2015). Fenofibrate, which is a peroxisome proliferator-activated receptor (PPAR) α agonist, is clinically used to lower lipid levels. Fenofibrate prevents diabetes-induced cardiac dysfunction, inflammation, and remodeling in streptozotocin-induced type 1 diabetic mice. Fenofibrate also increases the cardiac expression of *FGF21* and *sirtuin 1*. Diabetes-induced pathogenic effects in the heart are enhanced in *FGF21* knockout mice. Fenofibrate lowers the systemic lipid profile, but does not prevent heart deterioration in *FGF21* knockout diabetic mice. Exposure to high glucose levels significantly increases inflammatory responses, oxidative stress, and pro-fibrotic responses and also significantly inhibits autophagy in a cultured cardiomyocyte cell line. These effects are prevented by fenofibrate. This prevention is abolished by the inhibition of sirtuin 1. Thus, FGF21 prevents type 1 diabetes-induced pathological and functional cardiac abnormalities by up-regulating sirtuin 1-mediated autophagy (Zhang et al., 2016). These findings also indicate that FGF21 plays roles in cardiac protection in a paracrine manner. However, conflicting findings have shown that FGF21 is involved in cardiac protection in an endocrine manner as described below.

### Endocrine signal

#### FGF21

Myocardial ischemia with cardiomyocyte injury activates innate protective processes, which enhance myocardial tolerance to ischemia. In mice, FGF21 is released from hepatocytes and adipocytes into the circulation, and then contributes to myocardial protection through the mediation of the FGFR1/β-Klotho-PI3K-Akt1-BLC2-associated agonist of cell death (BAD) signaling network in an endocrine manner (Liu et al., 2013). Skeletal muscle is also an important site for the production of FGF21. The overexpression of *FGF21* by adenoviral vectors in the skeletal muscle of mice (Ad-*FGF21* mice) improves left ventricular systolic dysfunction and dilatation in a mouse model of myocardial infarction. Serum levels of adiponectin, which is a cardioprotective adipokine, are increased in Ad-*FGF21* mice. The beneficial effects of Ad-*FGF21* on cardiac dysfunction and inflammatory responses after myocardial infarction are diminished in *adiponectin*-knockout mice, indicating that muscle-derived FGF21 ameliorates adverse cardiac remodeling after myocardial infarction, at least in part, through an adiponectin-dependent mechanism (Joki et al., 2015).

Serum FGF21 levels are significantly decreased in streptozotocin-diabetic mice, however, no significant difference has been reported in blood glucose and triglyceride levels between *FGF21* knockout and control diabetic mice. *FGF21* knockout diabetic mice show earlier and more severe cardiac dysfunction, remodeling, and oxidative stress, as well as greater increases in cardiac lipid accumulation, which may contribute to increased cardiac oxidative stress and remodeling and the eventual development of diabetic cardiomyopathy (Yan et al., 2015).

Dietary methionine restriction (MR) mice exhibit hyperhomocysteinemia, which is a symptom associated with an increased risk of cardiovascular disease. However, an MR diet does not alter cardiac function in mice in spite of the presence of hyperhomocysteinemia. An MR diet induces the secretion of the cardioprotective hormones, adiponectin and FGF21, indicating that the adaptive responses of increased serum adiponectin and FGF21 levels protect against hyperhomocysteinemia (Ables et al., 2015).

## Pathophysiological roles of FGFs indicated by human diseases

FGF signaling disorders result in various human diseases including inherited diseases, metabolic diseases, and cancers (Carter et al., 2015; Ornitz and Itoh, 2015; Brewer et al., 2016; Degirolamo et al., 2016). Signaling disorders of FGF2, FGF21, and FGF23 as paracrine signals also result in heart diseases. In addition, heart diseases affect serum levels of FGF15/19, FGF21, and FGF23, indicating that these FGFs are serum biomarkers for heart diseases (Table 3).

**Table 3:** **Pathophysiological roles of FGFs in the human heart**.

	**Function**	**Effect**	**Disease**
**PARACRINE SIGNAL**			
FGF2	Gain-of-function	Promotion	Inflammatory pericardial disease
	Gain-of-function	Promotion	Type 4 cardiorenal syndrome
	Gain-of-function	Protection	TGFβ1-induced cardiac remodeling
FGF16	Loss-of-function	Promotion	Myocardial infarction and atrial fibrillation
FGF21	Gain-of-function		Atrial fibrillation with rheumatic heart disease
FGF23	Gain-of-function	Promotion	Left ventricular hypertrophy
	**Serum levels**		**Disease**
**SERUM BIOMARKER**			
FGF15/19	Decrease		Coronary artery disease
FGF21	Increase		Coronary artery disease
FGF23	Increase		Acute decompensated heart failure
	Increase		Kawasaki syndrome
	Increase		Left ventricular hypertrophy
	Increase		Oncostatin-dependent heart disease

### Paracrine signal

#### FGF2

Pericardial effusion is the abnormal accumulation of fluid in the pericardial cavity. Fluid accumulation leads to an increase in intrapericardial pressure, which has negative impacts on heart function. FGF2 levels in pericardial effusion are elevated in patients with inflammatory pericardial effusion. FGF2 has been suggested to participate in the pathogenesis of inflammatory pericardial disease (Karatolios et al., 2012).

Type 4 cardiorenal syndrome (CRS) refers to the cardiac injury induced by chronic kidney disease (CKD). Patients with type 4 CRS show elevated oxidative stress, which correlates with cardiac hypertrophy and a decreased ejection fraction. 5/6 subtotal nephrectomy rats mimic type 4 CRS. Cardiac FGF2, ERK1/2 phosphorylation, and nicotinamide adenine dinucleotide phosphate (NADPH) oxidase activity are significantly increased in 5/6 subtotal nephrectomy rats, suggesting that cardiac injury in type 4 CRS is mediated by an NADPH oxidase-dependent oxidative stress-activated ERK1/2 pathway and subsequent up-regulation of FGF2 (Liu et al., 2015).

Sustained elevations in pro-fibrotic TGFβ1 induces cardiac myofibroblast-mediated fibrosis and progressive structural tissue remodeling. Transforming growth factor (TGF) β1 significantly induces myofibroblast activation and extracellular matrix dysregulation. FGF2 attenuates TGFβ1-induced cardiac myofibroblast-mediated extracellular matrix remodeling in humans, suggesting that FGF2 prevents progressive maladaptive chamber remodeling and tissue fibrosis in patients with diverse structural heart diseases (Svystonyuk et al., 2015).

#### FGF16

*FGF16* loss-of-function mutations are linked to X-linked recessive hand malformations with fusion between the fourth and fifth metacarpals and hypoplasia of the fifth digit. Cardiac disorders including myocardial infarction and atrial fibrillation follow the *FGF16* mutated trait, indicating a relationship between the *FGF16* mutation and cardiac disease (Laurell et al., 2014).

#### FGF21

Although FGF21 is a typical endocrine signal, FGF21 in the heart may function as a paracrine signal as described above. Atrial fibrillation, which is the most common persistent arrhythmia, is a common manifestation of cardiovascular diseases including rheumatic heart disease. *FGF21* expression levels in atrial tissue and serum FGF21 levels are significantly increased in atrial fibrillation patients with rheumatic heart disease, indicating that FGF21 is involved in the development and maintenance of atrial fibrosis in atrial fibrillation with rheumatic heart disease in a paracrine manner (Wang R. et al., 2015).

#### FGF23

FGF23 typically regulates phosphate and vitamin D metabolism as a bone-derived hormone by mediating its biological responses in an FGFR-dependent manner. However, FGF23 binds to FGFRs with heparan sulfate with very low affinity. FGF23 efficiently binds to and activates FGFR1c with αKlotho as a co-factor (Erben, 2016). Although FGF23 is a typical endocrine signal, FGF23 in the heart may act as a paracrine signal as described below.

The levels of cardiac FGF23, which is expressed in cardiomyocytes, are excessively high in CKD patients. The enhanced myocardial expression of *FGF23* strongly correlates with the presence of left ventricular hypertrophy (LVH). Cardiac FGF23 levels are associated with the up-regulation of FGFR4 and activation of the calcineurin-NFAT signaling pathway, an established mediator of cardiac remodeling and LVH, indicating a strong relationship between LVH and elevated expression levels of FGF23, FGFR4, and calcineurin, as well as the activation of nuclear factor of activated T cells (NFAT) in the myocardium of patients with CKD (Leifheit-Nestler et al., 2016). FGF23 exclusively activates FGFR4 on human cardiomyocytes. A specific FGFR4-blocking antibody inhibits FGF23-induced hypertrophy in isolated cardiomyocytes and attenuates LVH in rats with CKD. *FGFR4* knockout mice do not develop LVH in response to elevated FGF23, whereas *FGFR4* knockin mice with an FGFR4 gain-of-function mutation spontaneously develop LVH. These findings indicate that FGF23 promotes LVH by activating FGFR4 in a paracrine manner (Grabner et al., 2015).

### Serum biomarkers

#### FGF15/19

FGF15/19 is a potential regulator of glucose and lipid metabolism, which may lead to atherosclerosis. Serum FGF15/19 levels are decreased in patients with metabolic syndrome, obesity, and non-alcoholic fatty liver disease (NAFLD) (Itoh et al., 2015; Zhang F. et al., 2015; Nies et al., 2016). Coronary artery disease (CAD) is an ischemic heart disease including stable angina, unstable angina, myocardial infarction, and sudden cardiac death. Serum FGF15/19 levels are negatively associated with the presence and severity of CAD, indicating that serum FGF15/19 levels are independent predictors of the extent of CAD (Hao et al., 2013).

#### FGF21

Serum FGF21 levels are significantly increased in CAD patients and are independently associated with adverse lipid metabolism (Lin et al., 2010). Serum FGF21 levels are also increased with NAFLD, metabolic disorders, and types 2 diabetes as well as CAD (Shen et al., 2013; Kim et al., 2015). However, by strictly matching body mass indices, serum FGF21 levels correlate in patients with metabolic disorders, but not CAD, indicating that this relationship is attributed more to preexisting cardio-metabolic factors than CAD *per se* (Lee et al., 2014; Domouzoglou et al., 2015).

#### FGF23

Serum FGF23 levels are also markedly elevated in patients with acute decompensated heart failure (ADHF), which is a sudden worsening of the signs and symptoms of heart failure, including difficulty breathing, leg or feet swelling, and fatigue (Andersen et al., 2016), with heart failure and a reduced ejection fraction (Koller et al., 2015), incident atrial fibrillation (Mathew et al., 2014), coronary heart disease, heart failure, and cardiovascular mortality (Lutsey et al., 2014), non-ischemic cardiac disease (Imazu et al., 2014), chronic systolic heart failure (Wohlfahrt et al., 2015), and CAD (Tuñón et al., 2016). However, myocardial *FGF23* gene expression levels and FGF23 immunostaining are not increased in ADHF patients, suggesting that the myocardium does not contribute to elevated circulating FGF23 levels in ADHF and that FGF23 plays roles in heart failure in an endocrine manner (Andersen et al., 2016).

Kawasaki syndrome is a vascular inflammatory disease that is associated with an increased risk of developing subsequent cardiac abnormalities in childhood. Patients often have elevated serum FGF23 levels with *FGF23* polymorphisms, which are correlated with cardiac abnormalities (Falcini et al., 2013; Masi et al., 2013).

Serum FGF23 levels, which are elevated with the progression of CKD, are associated with left ventricular hypertrophy (LVH), a major contributor to cardiovascular disease (Jimbo and Shimosawa, 2014; Wyatt and Drüeke, 2016). FGF23 causes pathological hypertrophy in isolated rat cardiomyocytes via the FGF receptor-dependent activation of the calcineurin-NFAT signaling pathway, which is independent of αKlotho. An intramyocardial or intravenous injection of FGF23 in mice results in LVH. In an animal model of CKD, a treatment with an FGF-receptor blocker attenuates LVH. The causal role for FGF23 in the pathogenesis of LVH suggests that chronically elevated serum FGF23 levels contribute directly to high rates of LVH and mortality in individuals with CKD (Faul et al., 2011; Faul, 2012).

Oncostatin M (OSM) is a major mediator of cardiac remodeling in heart failure. However, serum OSM levels are too low to serve as a suitable biomarker. FGF23 has been identified as a target of OSM in cultured cardiomyocytes. Serum FGF23 levels are increased in patients with myocarditis and ischemic and dilated cardiomyopathy. The macrophage/OSM axis is the major mechanism of cardiac FGF23 expression and release into the circulation in heart failure, indicating that FGF23 as a paracrine signal is a promising biomarker of OSM-dependent heart diseases (Richter et al., 2015).

### Therapeutic potential

Although the therapeutic potential of FGFs in cardiac disorders has been indicated by animal models described above, it has not been reported in humans. However, the findings by animal models provide therapeutic strategies for cardiac disorders in humans.

## Roles of FGFs in stem cell biology

### Embryonic stem cells and induced pluripotent stem cells

Embryonic stem (ES) and induced pluripotent stem (iPS) cells have multipotency to differentiate into various cell lineages (Takahashi and Yamanaka, 2015). Cells and tissues differentiated from ES and iPS cells have potential as a novel therapy for advanced heart failure (Liu et al., 2016; Terzic and Behfar, 2016). FGF2 and FGF10 enhance the cardiomyogenic differentiation of stem cells, indicating that FGF signaling is useful for stem cell therapy for heart failure.

#### FGF2

The combination of FGF2 and BMP2 efficiently enhances the cardiomyogenic differentiation of ES cells at an optimal concentration for the first 3 days. The inhibition of FGF2 markedly suppresses cardiomyogenic differentiation, indicating that FGF signaling plays a crucial role in early cardiomyogenesis (Kawai et al., 2004). FGF2 and BMP2 also induce iPS cell differentiation into cardiomyocytes (Yamasaki et al., 2013). The achievement of efficient cardiac differentiation using FGF2 may facilitate ES or iPS cell-derived cell therapy for heart diseases.

#### FGF10

FGF10 also promotes cardiomyocyte differentiation from ES and iPS cells. In addition, co-administration of FGF10 and ES cells in the myocardium promotes cardiomyocyte differentiation in the heart (Chan et al., 2010).

### Direct reprogramming from fibroblasts

One cell type may be directly converted into another without reverting to a stem cell state by overexpressing lineage-specific factors. Direct reprogramming has been proven sufficient for yielding a diverse range of cell types from fibroblasts, including cardiomyocytes, endothelial cells, hematopoietic cells, and stem/progenitor cells (Sadahiro et al., 2015). Direct reprogramming is also potentially useful as a novel therapy for advanced heart failure.

#### FGF2 and FGF10

Fibroblasts may be directly reprogrammed into cardiomyocyte-like cells by the overexpression of cardiac transcription factors, however, this process is inefficient under serum-based culture conditions. The combination of FGF2, FGF10, and vascular endothelial growth factor promotes cardiac reprogramming under defined serum-free conditions. The combination of these secreted signals activates multiple cardiac transcriptional regulators and converts partially reprogrammed cells into functional cardiomyocyte-like cells through the RAS-MAPK and PI3K/AKT pathways (Yamakawa et al., 2015).

## Conclusions

FGFs are paracrine growth factors, hormone-like endocrine factors, and intracrine factors with diverse functions in development, health, and disease. Findings obtained from mouse models and human diseases with FGF signaling disorders have indicated that several FGFs are involved in heart development, health, and disease. FGF8, FGF9, FGF10, and FGF16 function as paracrine signals in embryonic heart development. In addition, FGF2, FGF9, FGF10, and FGF16 play roles as paracrine signals in postnatal heart pathophysiology. Although FGF15/19, FGF21, and FGF23 are endocrine FGFs, they also mostly act as paracrine signals in heart development or pathophysiology. Serum FGF15/19 levels are decreased and FGF21 and FGF23 levels are increased in heart diseases, which also suggests their roles in heart pathophysiology. In addition, FGF2 and FGF10 stimulate the cardiac differentiation of cultured stem cells and cardiac reprogramming of cultured fibroblasts. These findings provide new insights into the roles of FGF signaling in the heart and potential therapeutic strategies for cardiac disorders.

## Author contributions

NI, making an original plan and writing a manuscript. HO, writing a manuscript and discussing it. YN, writing a manuscript and discussing it. MK, writing a manuscript and discussing it.

### Conflict of interest statement

The authors declare that the research was conducted in the absence of any commercial or financial relationships that could be construed as a potential conflict of interest.
